# Association of metformin administration with gut microbiome dysbiosis in healthy volunteers

**DOI:** 10.1371/journal.pone.0204317

**Published:** 2018-09-27

**Authors:** Ilze Elbere, Ineta Kalnina, Ivars Silamikelis, Ilze Konrade, Linda Zaharenko, Kristine Sekace, Ilze Radovica-Spalvina, Davids Fridmanis, Dita Gudra, Valdis Pirags, Janis Klovins

**Affiliations:** 1 Latvian Biomedical Research and Study Centre, Riga, Latvia; 2 Riga East Clinical University Hospital, Riga, Latvia; 3 Department of Endocrinology, Pauls Stradins Clinical University Hospital, Riga, Latvia; 4 University of Latvia, Faculty of Biology, Riga, Latvia; Rush University, UNITED STATES

## Abstract

**Background:**

Metformin is a widely used first-line drug for treatment of type 2 diabetes. Despite its advantages, metformin has variable therapeutic effects, contraindications, and side effects. Here, for the very first time, we investigate the short-term effect of metformin on the composition of healthy human gut microbiota.

**Methods:**

We used an exploratory longitudinal study design in which the first sample from an individual was the control for further samples. Eighteen healthy individuals were treated with metformin (2 × 850 mg) for 7 days. Stool samples were collected at three time points: prior to administration, 24 hours and 7 days after metformin administration. Taxonomic composition of the gut microbiome was analyzed by massive parallel sequencing of *16S rRNA* gene (V3 region).

**Results:**

There was a significant reduction of inner diversity of gut microbiota observed already 24 hours after metformin administration. We observed an association between the severity of gastrointestinal side effects and the increase in relative abundance of common gut opportunistic pathogen *Escherichia-Shigella* spp. One week long treatment with metformin was associated with a significant decrease in the families *Peptostreptococcaceae* and *Clostridiaceae*_1 and four genera within these families.

**Conclusions:**

Our results are in line with previous findings on the capability of metformin to influence gut microbiota. However, for the first time we provide evidence that metformin has an immediate effect on the gut microbiome in humans. It is likely that this effect results from the increase in abundance of opportunistic pathogens and further triggers the occurrence of side effects associated with the observed dysbiosis. An additional randomized controlled trial would be required in order to reach definitive conclusions, as this is an exploratory study without a placebo control arm. Our findings may be further used to create approaches that improve the tolerability of metformin.

## Introduction

Metformin is a biguanide agent that is widely used as a first-line treatment of type 2 diabetes (T2D) [1]. Metformin has several advantages, including high safety indicators, high efficacy, neutral or lowering effect on body mass, and cardioprotective effects [2–4], resulting in broad indications for use over the 60 years it has been on the market. Nevertheless, metformin also has variable therapeutic effects, contraindications, and side effects which indicate the urgent need for a personalized approach when choosing treatment strategies [5].

It has been shown that intravenously administered metformin is less effective than its orally administered form [6]. Furthermore, metformin reaches a 30–300 times higher concentration in mucosa of small intestine compared to plasma, and up to 30% of the drug is eliminated through the feces [7, 8]. In addition, a delayed-release formulation of metformin improves glycemic control to the same extent as the immediate-release form despite lower systemic exposure [9]. These findings have led to the hypothesis that the effects of metformin are partially explained by its interaction with the gut microbiome. The connection between the effects of metformin and the gut microbiome has been supported by several recent studies [10–17]. These studies suggest that the gut microbiome is involved in both the therapeutic and side effects of the drug, yet details of this interaction remain obscure.

Current knowledge regarding the interaction between metformin and the gut microbiome highlights that metformin reduces inner diversity of the gut microbiome in mice fed a high-fat diet [13] and its administration increases relative abundance of *Akkermansia muciniphila* [10–14]. There is also evidence that metformin increases the abundance of some other mucin degrading and short-chain fatty acids producing genera [10], as well as opportunistic pathogens such as *Escherichia* spp. [11, 12]. Modulation of the gut microbiome is also hypothesized to be responsible for the anti-obesity action of metformin, not only in T2D patients but in pre-diabetic populations as well [18].

However, as pointed out previously, many of the earlier studies of the gut microbiome did not control for treatment regimens in T2D patients, subsequently leading to divergent conclusions [11]. It appears plausible that some of the potential clinical effects, e.g., metabolic control of longevity [19], anticancer properties [20], and testosterone lowering in patients with polycystic ovary syndrome [21] occur through alterations in the microbiome. Therefore, in this exploratory longitudinal study we evaluated the short-term effect of oral metformin administration on the human gut microbiome composition and diversity in healthy individuals, and the possible connection between these changes and metformin-related gastrointestinal (GI) side effects.

## Materials and methods

### Study design

**Fig 1:**
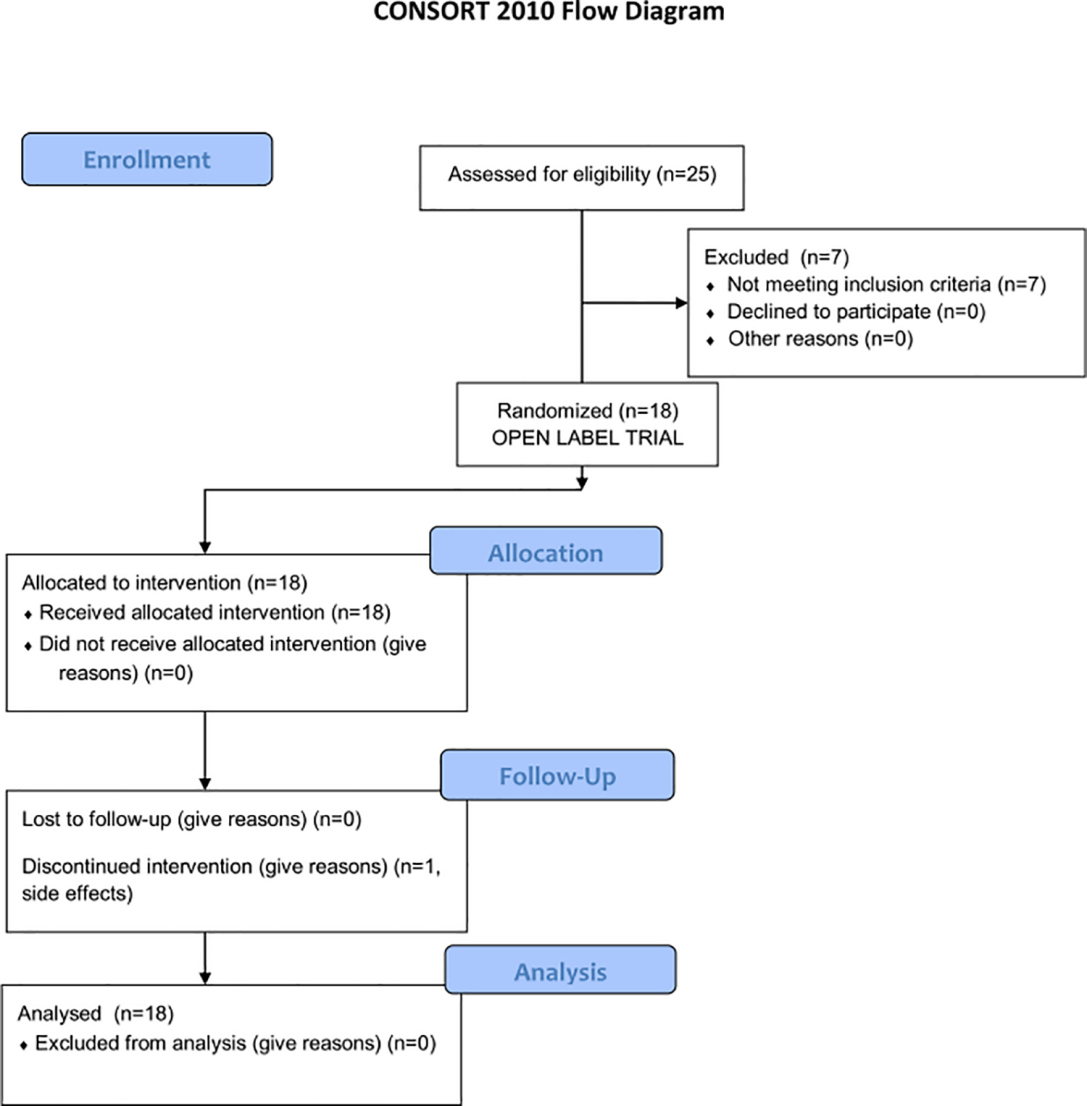
CONSORT flowchart of the open-label trial.

Eighteen healthy volunteers of Caucasian origin were included in this exploratory study through the Genome Database of Latvian Population [22] as a part of an ongoing clinical trial (50 individuals to be included in total), by assessing the 25 individuals available at the time. Baseline characteristics and registered clinical parameters are shown in Table 1. Major exclusion criteria were as follows: (1) use (during the past two months) of antibiotics, immunosuppressive drugs, corticosteroids, proton pump inhibitors, or pharmaceutical-grade probiotics; (2) oncological, autoimmune, or chronic gastrointestinal tract diseases, or T2D; (3) diarrhea in the past week; and (4) use of any other medications that are not compatible with metformin. A full list of inclusion/exclusion criteria can be found in the S1 Text. All participants, after full explanation of the purpose and nature of all procedures used, gave signed informed consent containing detailed information on the project (Fig 1). The study was carried out in accordance with the Declaration of Helsinki, and approved by the Central Medical Ethics Committee (1/16-05-12) and State Agency of Medicines of the Republic of Latvia (17–1723), clinical trial registration number: 2016-001092-74 (www.clinicaltrialsregister.eu).

**Table 1:** Characteristics of the study group.

Characteristic	Value
Females/Males, n (%)	11 (61.1%)/ 7 (38.9%)
Age (years), median [IQR]	25.5 [7.5]
BMI, median [IQR]	24.2 [3.5]
ALAT (U/l), median [IQR]	20.5 [10.8]
Creatinine (μmol/l), median [IQR]	71.5 [13.5]
Fasting plasma glucose (mmol/l), median [IQR]	5.1 [0.5]

ALAT–alanine aminotransferase, BMI–body mass index, IQR–interquartile range

Participants took metformin (850 mg tablets; Berlin-Chemie AG, Germany) twice daily during meals with a glass of water for a period of 7 days. Diet, physical activities, and side effects were registered daily in special questionnaires during the whole study period. Dietary data were registered using a 7-day food record during the week of metformin use, and an additional 2-day food record was filled before starting the use of metformin. We consulted a certified nutritionist and data from the dietary registry were divided into 11 food groups and labeled as follows: (1) milk and dairy products, (2) vegetables, (3) fruits, (4) meat and its products, (5) fish, (6) croppers, (7) nuts and seeds, (8) fat, (9) snacks, (10) sweetened drinks, and (11) alcohol. The cumulative summary characterizing the 7-day food records for each food group was expressed as a percentage from the combined amount of food consumption during the metformin treatment (S2 Table).

The primary endpoint of this study was the detection of significant changes in taxonomical composition of the gut microbiome. The secondary endpoint was the possible correlation between specific taxonomic units and the development of GI side effects. Compliance with the study was ensured by thorough explanation and detailed written instructions of the study protocol. Unused tablets were returned to the principal investigator.

All individuals were concurrently involved in an ongoing methylation profile analysis in leukocytes from whole blood samples taken at three specific time points during the study (unpublished data).

### Sample collection

Blood samples for hematological and biochemical analyses were collected in the fasting state 1–3 days before starting metformin administration. Data were used to evaluate significant health indicators for kidney and liver function, as well as other criteria characterizing the suitability of individuals for medicament therapy. All hematological and biochemical analyses were conducted in the same certified clinical laboratory.

Stool samples in two aliquots were collected at three time points: before starting metformin treatment (M0) and 24 hours (M24h) and 7 days (M7d) after the first intake of metformin. After collection, fecal samples were stored at room temperature until delivery to the laboratory, and frozen at −80°C as soon as possible but not later than within 24 hours of collection [23, 24]. Sample collection, storage and handling were done by following our developed standard operation procedures with the aim to minimize unnecessary freezing and thawing cycles and to reduce the possibility of artefacts caused by temporary storage at room temperature.

### Bacterial DNA preparation and sequencing analysis

Microbial DNA was extracted from frozen stool samples using FastDNA Spin Kit for Soil (MP Biomedicals, Santa Ana, CA, USA) and FastPrep Instrument according to the instructions of the manufacturer. DNA concentrations of the extracted samples were evaluated using Qubit 2.0 fluorometer (Thermo Fisher Scientific, Waltham, MA, USA), and the integrity of the extracted microbial DNA was validated by agarose gel electrophoresis.

For each sample, the V3 region of the *16S rRNA* gene was amplified using the Probio_Uni/Probio_Rev primer set [25]. Each primer contained IonXpress adapter sequence and a unique barcode sequence. The amplified PCR products were purified using NucleoMag magnetic beads (Macherey-Nagel, Düren, Germany), and their quantity and quality were evaluated with the Agilent 2100 Bioanalyzer DNA High Sensitivity chip (Agilent Technologies, Santa Clara, CA, USA). Sequencing of the amplicon libraries was performed with Ion Torrent Personal Genome Machine (PGM) System (Thermo Fisher Scientific; Ion 318 Chip Kit v2, Ion PGM Hi-Q Sequencing Kit, minimal sequencing depth per sample– 250 000 reads) according to the instructions of the manufacturer.

### Preprocessing and statistical methods

Raw sequence data were processed using mothur software v.1.39.1 [26]. Analyses were done using a modified version of the publicly accessible MiSeq SOP. In the sequence filtering, step reads were removed if they were 75 bp or shorter, or contained ambiguous bases or homopolymers longer than eight bases. A representative sequence from each cluster was chosen and used to identify taxonomic groups from the SILVA database v.123 [27]; the flip parameter was set as true. Chimeric sequences and sequences containing potential sequencing errors were removed using UCHIME [28] or pre-clustering (threshold = 2), respectively. Operational taxonomic units were defined at ≥99% sequence identity, using the OptiClust algorithm. Reads were classified using the naïve Bayesian classifier [29].

The correlation between gut microbiome taxa and the defined food groups was evaluated with Spearman’s correlation analysis and the results were adjusted for multiple testing using the Benjamini–Hochberg method.

Statistical analyses were performed on taxonomic units found in at least 50% of samples with R program v.3.2.2 packages edgeR, limma, phyloseq, DESeq, vegan (adjustment for multiple testing by Benjamini–Hochberg method), and graphics were created with package ggplot2. Sample normalization was done as implemented in edgeR (calcNormFactors function) or the relative abundances were used if necessary. Additional analysis to detect differential abundance was performed using the Linear discriminant analysis Effect Size (LEfSe) method [30] integrated in the Galaxy framework. In particular, the non-parametric Kruskal–Wallis sum-rank test was used to detect differentially abundant taxa, and Linear Discriminant Analysis (LDA) was used to estimate the effect size. The genus level alpha diversity of each sample was calculated by the Shannon index [31], beta diversity across samples was evaluated with non-metric multidimensional scaling (NMDS) using Bray–Curtis distances. Permutational multivariate analysis of variance (PERMANOVA) was used (permutations = 9999) for comparing the analyzed groups of ordinations. Statistical significance for changes of Shannon index and for taxonomic units between specific sample groups was evaluated by Wilcoxon signed-rank test.

## Results

### Main characteristics of the samples

In total 53 stool samples were obtained from 18 healthy individuals. All characteristics depicted in Table 1, except for age and ALAT, corresponded to the Gaussian distribution. One participant withdrew from the trial at the fifth day of metformin administration due to severe GI side effects. The stool sample from this individual was collected after five days long metformin administration, and during the analysis it showed high similarity to all other M7d samples, so it was further analyzed together with this group.

After evaluation of registered side effects, we divided individuals into three groups according to the severity of GI side effects observed during the metformin administration: (1) no side effects (n = 3); (2) mild side effects defined by meteorism, stomach ache, nausea, and loss of appetite (n = 6); and (3) severe side effects defined by loose stools 1–3 times a day, diarrhea, and vomiting (n = 9). Only four individuals had loose stools (1–2 times per day) on day 1 of the study. The average time of occurrence for severe side effects was the day 3 of treatment. Full description on the registered adverse events can be found in S1 Table.

**Fig 2:**
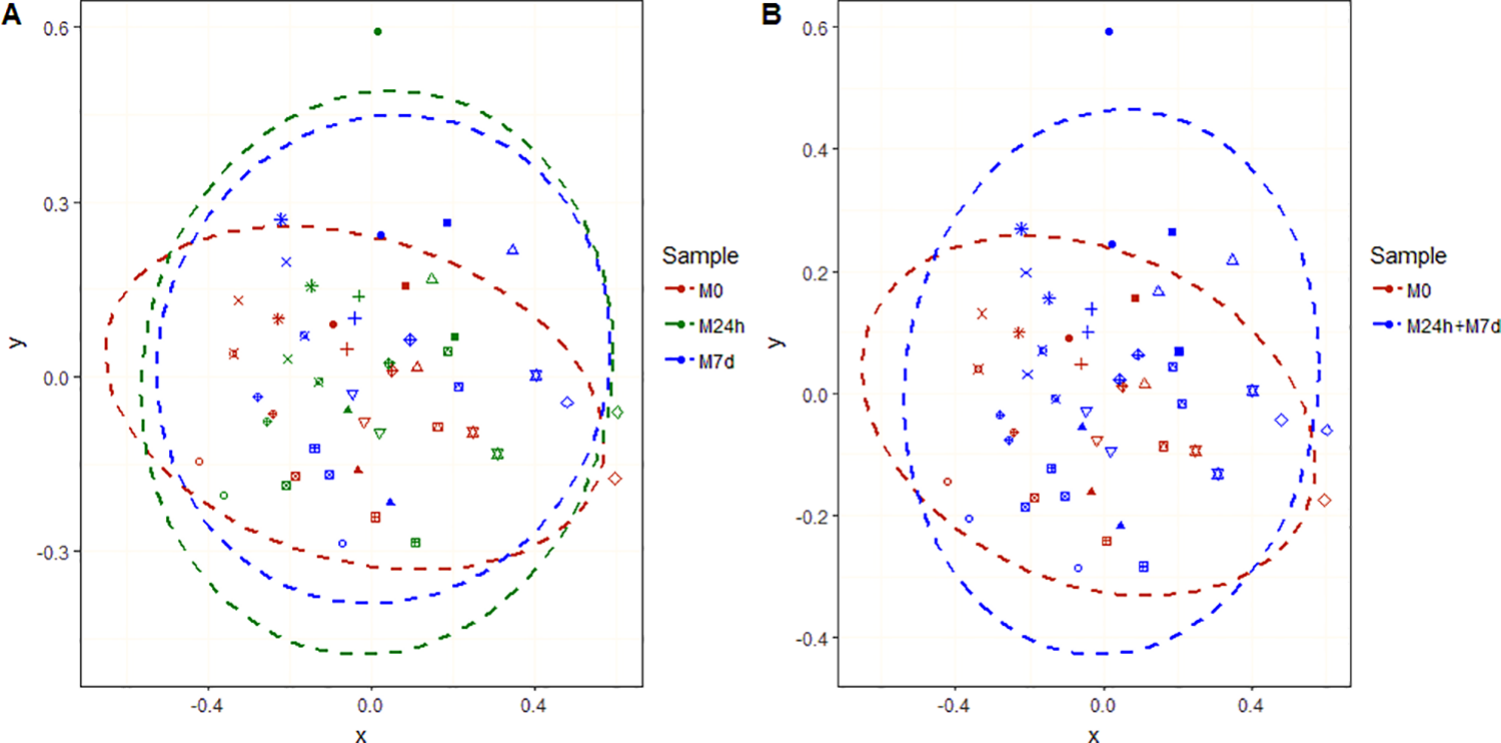
NMDS plots representing diversity between samples at genus level based on Bray–Curtis distances. (A) Comparison between all sample groups. (B) Comparison between M0 sample and samples during metformin administration (M24h + M7d). Ellipses represent the 95% confidence interval surrounding each group of samples. Different symbols represent participants of the study.

To evaluate the general differences in gut microbiota between the control sample and the samples taken after metformin administration we performed ordination analysis (Fig 2A and 2B) based on Bray–Curtis distances. As expected, gut microbiome communities were specific to each individual (PERMANOVA: R^2^ = 0.74, p = 0.001) (S1 Fig). Thus, for further comparison of ordinations we used each individual as a nested factor. The analysis did not show any significant difference between the three groups of samples as defined by time points (M0, M24h, and M7d) (PERMANOVA: R^2^ = 0.028, p = 0.078). Merging together both of the sample groups collected during and after metformin administration (M24h and M7d) and comparison with the control sample (M0) revealed a significant difference (PERMANOVA: R^2^ = 0.019, p = 0.036).

### Metformin reduces inner diversity of the gut microbiome

**Fig 3:**
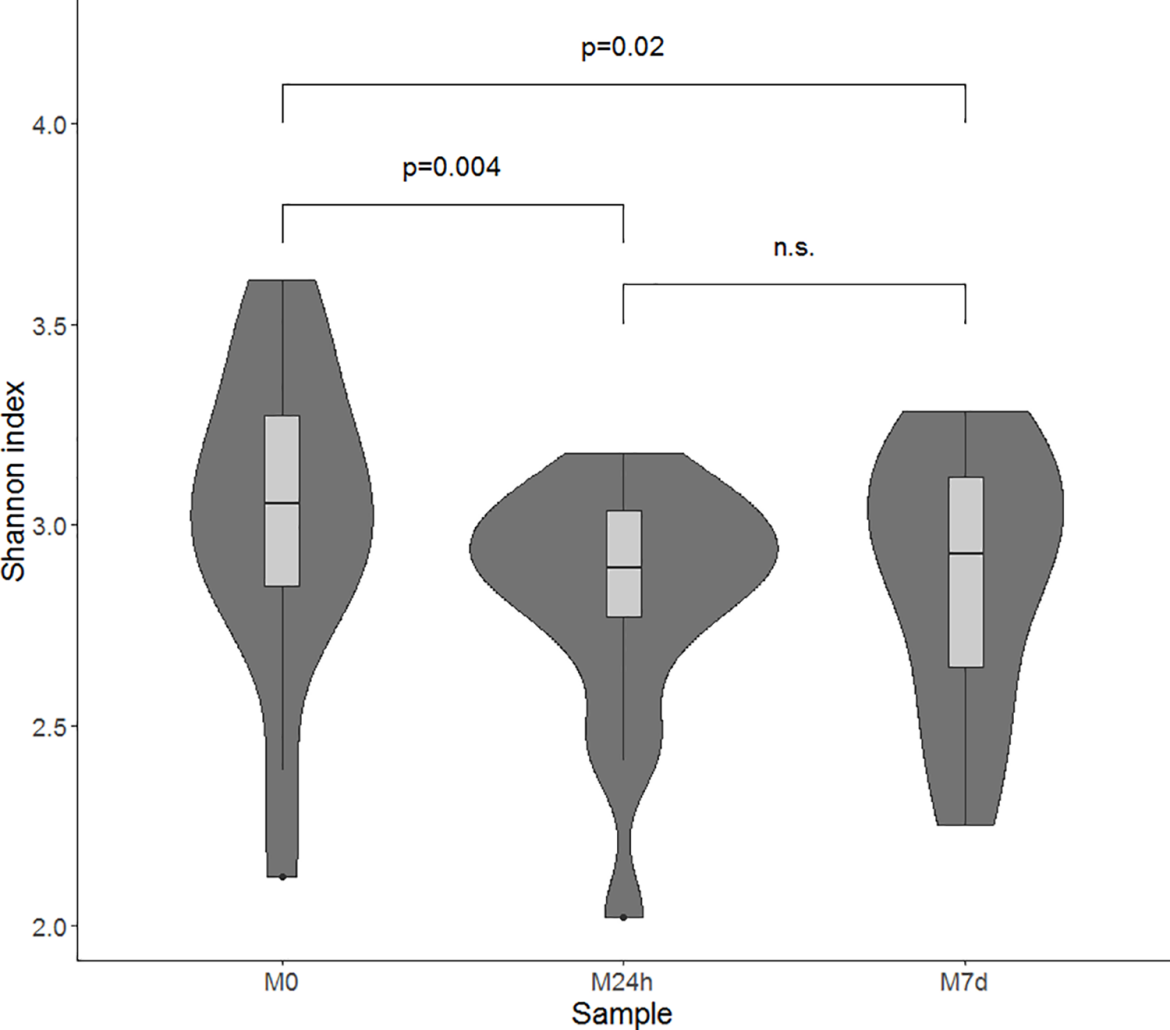
Alpha diversity changes during metformin therapy, evaluated at different time points. Samples marked as follows: M0—before starting metformin treatment; M24h - 24 hours after first intake of metformin; M7d - after 7 days treatment with metformin. Violin plot characterizing Shannon indexes combines boxplots, representing the median value and interquartile ranges, with kernel density plots.

Comparing the Shannon index between the groups (Fig 3) we found that metformin therapy significantly reduces inner diversity of the gut microbiome immediately after the first two or three doses of metformin. After 7 days of metformin administration the inner diversity of the gut microbiome in study participants slightly increased, but was still significantly lower than before the use of metformin.

### Changes in abundance of opportunistic pathogens in groups with different severity of GI side effects

To determine if the reduced inner diversity of the microbiome was associated with further gut microbiome dysbiosis, we analyzed changes in the abundance of common gut opportunistic pathogen *Escherichia-Shigella* spp. We used the Wilcoxon-rank test for targeted analysis of possible changes in the relative abundance of *Escherichia-Shigella* genus comparing the three time points. There was no significant changes observed between the M0 (MED = 0.03%; IQR = 0.37%) and M24h ((MED = 0.05%; IQR = 0.14%) or M7d (MED = 0.46%; IQR = 1.04%). The relative abundance of these opportunistic pathogens was increased in the M7d sample when compared to M24h sample.

**Fig 4:**
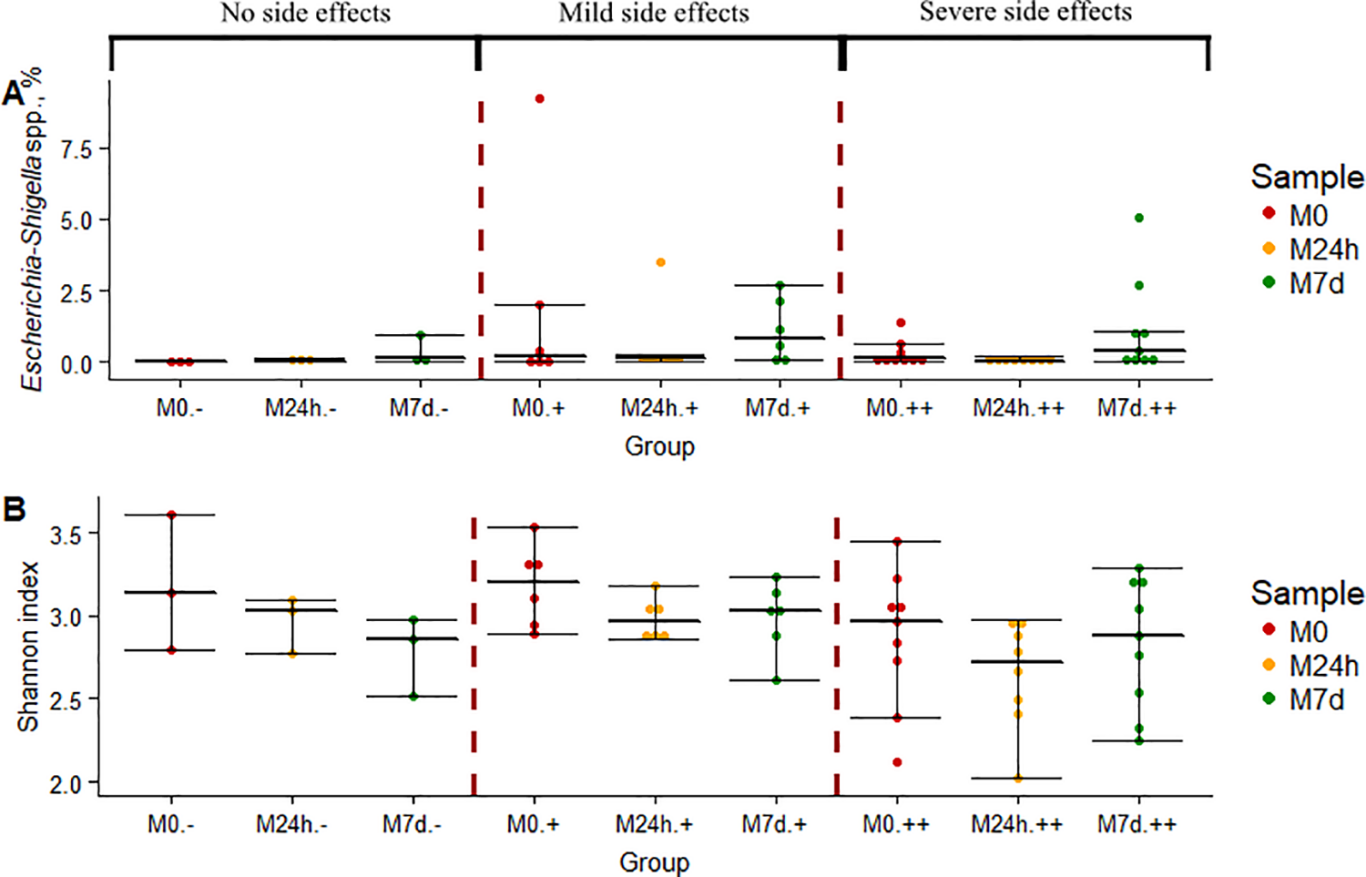
Changes in gut microbiome alpha diversity and abundance of opportunistic pathogen *Escherichia-Shigella* spp. at different time points within groups defined according to severity of GI side effects. (A) Changes in the relative abundance of *Escherichia-Shigella* spp. (B) Inner diversity changes, characterized by Shannon index. Samples marked as follows: M0—before starting metformin treatment; M24h - 24 hours after first intake of metformin; M7d - after 7 days treatment with metformin. Groups defined by observed side effects: “–”no side effects (n = 3), “+” mild side effects (n = 6), “++” severe side effects (n = 9). Dot plots depict median, 25th percentile and 75th percentile of data in each group. Dots beyond the bounds of the whiskers represent outliers.

In order to test the possible relation of these changes with observed side effects we compared the changes in relative abundance of *Escherichia-Shigella* spp., as well as overall alpha diversity in different GI side effect categories (Fig 4). The inner diversity in the M7d sample compared to M24h sample increased only in groups with side effects. Thus in the group with mild side effects the median Shannon index for M7d sample was 3.03 (IQR = 0.21) compared to 2.97 (IQR = 0.15) in M24h sample, while in the group with severe side effects median was 2.88 (IQR = 0.66) for M7d sample compared to 2.72 (IQR = 0.42) for the M24h sample. We also observed increased presence of *Escherichia-Shigella* spp. in the samples taken before metformin administration from the participants later experiencing mild or severe side effects with the following median values of 0.21% (IQR = 1.57%) and 0.13% (IQR = 0.33%) respectively. The presence of *Escherichia-Shigella* spp. in the group with no side effects was beyond detectable limits.

### Differential abundance of taxonomic groups

To observe in-depth changes in the composition of the gut microbiome we used edgeR and evaluated the statistical significance of differential abundance of taxonomic groups between time points at every taxonomical level (phylum, class, order, family and genus). In total, 220 taxonomic groups presented in at least 50% of samples were tested. The main results are summarized in Table 2. There were no significant changes in representation of taxonomic groups at the phylum level at any of the contrasts between the M0, M24h, and M7d samples. One week treatment with metformin was associated with significant decreases in the families *Peptostreptococcaceae* and *Clostridiaceae_1* and four genera within these families: *Peptostreptococcaceae*_unclassified (family *Peptostreptococcaceae*), *Clostridiaceae_1*_unclassified (family *Clostridiaceae_1*), *Asaccharospora* (family *Peptostreptococcaceae*), and *Romboutsia* (family *Peptostreptococcaceae*). Comparison of the M24h and M7d samples showed significantly increased abundance of the order *Enterobacteriales*, including the only family in this order–*Enterobacteriaceae* with the genus, *Escherichia-Shigella*.

**Table 2:** Main significant changes in taxonomic units at all taxonomic levels.

Taxonomic level	Taxonomic group	Average abundance in sample groups,%			P–value [FDR*]		
		M0	M24h	M7d	M0 vs. M24h	M24h vs. M7d	M0 vs. 7d
**Class**	*Proteobacteria* unclassified	0.019	0.008	0.02	0.03 [0.62]		
	*Gammaproteobacteria*	1.16	0.50	1.71		0.002 [0.05]	0.008 [0.13]
	*Verrucomicrobiae*	0.45	0.30	1.14		0.03 [0.20]	
	*Bacilli*	1.02	0.83	1.31		0.03 [0.20]	0.04 [0.17]
	*Epsilonproteobacteria*	0.003	0.007	0.01			0.01 [0.13]
	*Negativicutes*	2.38	1.90	1.34			0.02 [0.15]
	*Proteobacteria*_unclassified	0.02	0.008	0.02	0.02 [0.68]		
	*Enterobacteriales*	0.99	0.41	1.55		**0.002 [0.04]**	0.005 [0.12]
**Order**	*Verrucomicrobiales*	0.45	0.30	1.14		0.03 [0.26]	
	*Lactobacillales*	1.00	0.81	1.29		0.03 [0.26]	0.04 [0.36]
	*Selenomonadales*	2.38	1.90	1.34			0.02 [0.26]
	*Peptostreptococcaceae*	1.17	0.93	0.23		**0.001 [0.02]**	**4.24E-06 [0.0002]**
	*Clostridiaceae*_1	0.70	0.51	0.13		**0.008 [0.12]**	**3.41E-05 [0.0007]**
**Family**	*Enterobacteriaceae*	0.99	0.41	1.55		**0.001 [0.02]**	0.004 [0.05]
	*Streptococcaceae*	0.58	0.41	0.68		0.01 [0.14]	
	*Verrucomicrobiaceae*	0.45	0.30	1.13		0.03 [0.21]	
	*Peptostreptococcaceae*_unclassified	0.91	0.72	0.18	0.04 [0.97]	**0.0006 [0.04]**	**1.86E-06 [0.0002]**
	*Clostridiaceae*_1_unclassified	0.63	0.49	0.10		0.032 [0.08]	**8.40E-06 [0.0005]**
	*Asaccharospora*	0.17	0.15	0.03		0.003 [0.08]	**1.64E-05 [0.0006]**
	*Romboutsia*	0.09	0.06	0.02		0.002 [0.07]	**2.92E-05 [0.0009]**
	*Escherichia-Shigella*	0.80	0.27	1.00		**0.0006 [0.04]**	0.008 [0.14]
**Genus**	*Streptococcus*	0.45	0.35	0.61		0.007 [0.16]	0.02 [0.31]
	*Enterobacteriaceae*_unclassified	0.19	0.13	0.48		0.01 [0.19]	0.004 [0.11]
	*Ruminiclostridium*_6	0.45	0.35	0.08		0.03 [0.45]	0.006 [0.13]
	*Akkermansia*	0.44	0.30	1.13		0.03 [0.48]	
	*Ruminococcaceae*_UCG-008	0.02	0.03	0.04			0.01 [0.16]
	*Blautia*	1.45	2.04	2.02			0.04 [0.52]

* Tendencies that maintained significance after false discovery rate (FDR) correction are marked in bold.

**Fig 5:**
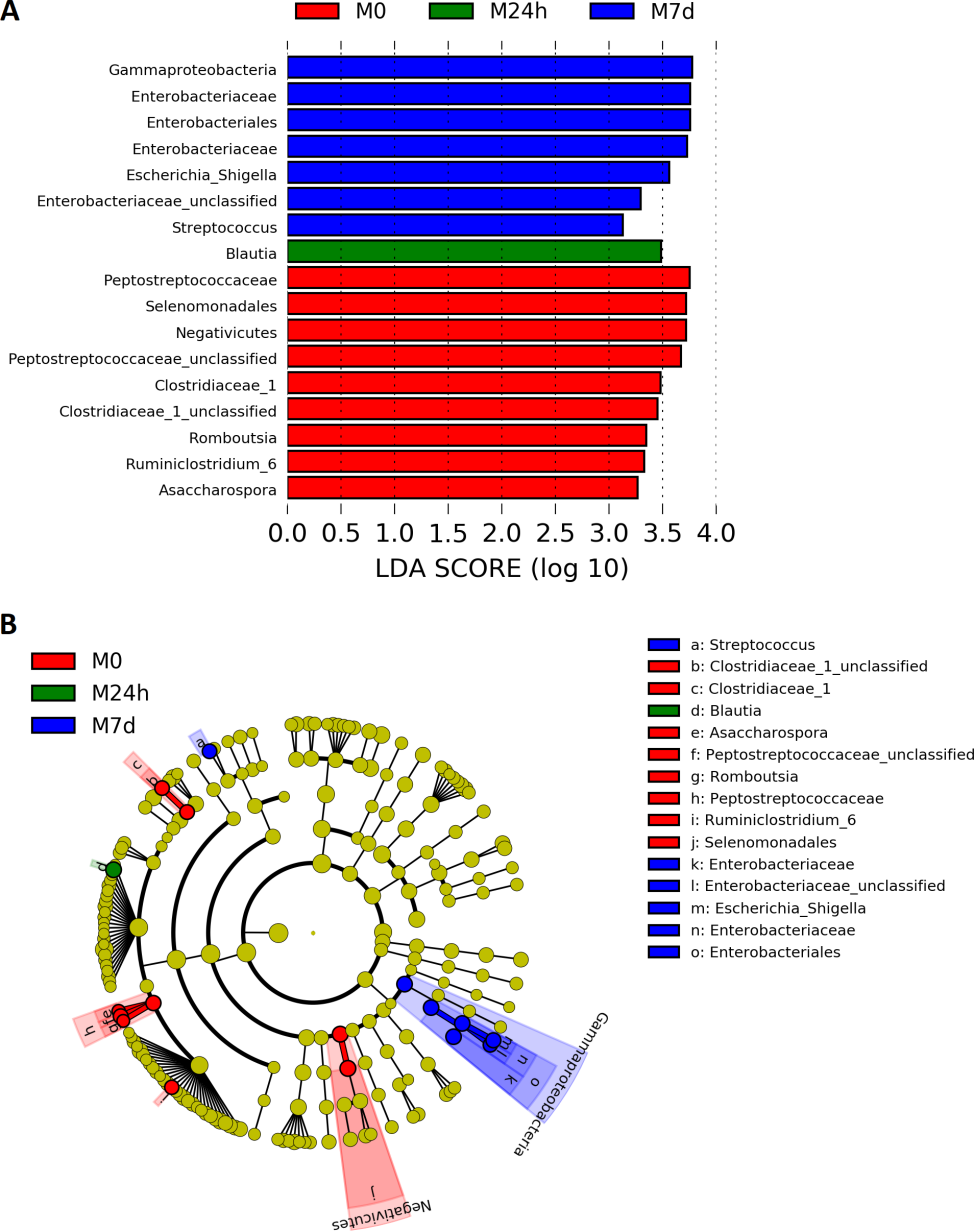
Comparison of LDA effect size of the significantly differentiating microbial taxa deduced using LefSe analysis. (A) Differences in abundance of taxonomic groups among all three sample. LDA cutoff = 2. Differentiating feature analysis was carried out with Kruskal–Wallis test raw p-value cutoff = 0.05. (B) Cladogram illustrating the phylogenetic relationship among the significantly differentiating gut microbiome taxonomic groups among the M0, M24h, and M7d samples.

In addition, for graphic representation of differentially abundant taxa as well as their effect sizes and phylogenetic relationship, the LEfSe method was performed (Fig 5). This method detected 17 differentially abundant taxonomic clades, which mainly matched with those found with edgeR analysis.

In order to verify the findings from previous publications reporting that metformin increased abundance of *Akkermansia* spp., we performed a targeted Wilcoxon–rank test. Comparisons between two pairs were significant: M0 vs. M7d (p = 0.03) and M24 vs. M7d (p = 0.01) but the significance disappeared after performing the correction for multiple testing. This genus was present in 37 of 53 samples, but the tendency and direction of changes in abundance were not consistent in all individuals for this taxa.

In conclusion, to evaluate the possible confounding effect of diet, Spearman correlation analysis was carried out. We did not find any significant association between the changed taxa and our defined food groups after performing the correction for multiple testing.

## Discussion

In order to observe unbiased short-term effects of metformin on the gut microbiome we used an exploratory longitudinal study design and included healthy individuals. We believe that this design should have minimized false associations and conclusions arising from unaccounted treatment status by metformin or other medications in T2D patients, including the unknown true duration of T2D before diagnosis and the high interindividual variation of the gut microbiome. It has been recognized that, in similar time series studies, individuals can be treated as their own controls before and during treatment [32]. In addition, the strong effect size in previously described metformin studies [12] allowed us to consider the longitudinal study design as sufficiently powerful to achieve the goal of our study. Taking in account our results, this study design has as well prevented any confounding effects induced by the known high interindividual variety of diet [33], as we did not find any significant association between the changed taxonomic composition and data from the 7-day food record. Furthermore, there has been an increase in the use of metformin beyond diabetes, so this research may give additional insights into general features of the interaction between metformin and the gut microbiome, which may be further applicable to its use across a broad range of diseases, such as Alzheimer’s disease [34], polycystic ovary syndrome [21], various types of cancers [20], and prevention of diabetes in individuals with prediabetic symptoms [35]. We also chose to include the first sampling point 24 hours after metformin administration in order to observe the effects as soon as possible and to avoid the potentially strong influence of diarrhea and other side effects known to occur after metformin administration. The generally accepted incidence of metformin-induced GI side effects is 20 – 30% [36, 37]. However, our data agreed with recent reports [38, 39], as we observed a high rate of side effects in our study (50% of study group experienced strong and 33% experienced mild side effects). This could be explained by the rather high initial dose of metformin, or the possibility that the design of the recent studies was more feasible for patients, which ensured higher treatment adherence and higher rate of reporting side effects.

Our findings that show the reduction in inner diversity of the gut microbiome during metformin treatment was in line with the previously observed effects of metformin effects in mice and rat models [13, 17]. In addition, a recent study using metagenome sequencing showed that metformin improves microbial gene richness among T2D patients, while metformin users generally have lower gene richness than healthy controls have [11]. It should be noted that, in our case, the reduction of diversity was observed at the markedly short time period of 24 hours, in the absence of diarrhea (only four participants experienced loose stools on the day 1). The small increase in inner diversity when comparing the M24h and M7d samples indicates the tendency of the gut microbiome to regain its ecological equilibrium even in participants experiencing diarrhea, as seen in the group of participants with severe side effects, in which nine people experienced loose stools or diarrhea. Likewise, this explains the growth of opportunistic pathogens including members from genus *Escherichia-Shigella* spp., which in previous studies has been associated with metformin treatment in T2D patients [11, 12]. Although, we cannot attribute the rapid increase of this genus between 24-hour and 7-day time points as a direct effect of metformin, this effect could be ensured by the trait of persistence of this genus [40] and high abilities to adapt [41]. Therefore, it can occupy the space open due to unfavorable conditions created by yet fully unknown effect of metformin. In other words, the reduced diversity in the gut presents *Escherichia-Shigella* spp. the free niche needed to emerge in larger numbers compared to the concurrent bacterial species. The connection between reduced alpha diversity and the further increase in the representation of opportunistic pathogens has been described before in the context of antibiotic treatment, various diseases and aging [11, 42–45].

The characteristic GI side effects in most cases manifest at the beginning of metformin therapy and usually disappear after several weeks [46, 47]. Several species from *Escherichia-Shigella* spp. have been identified as pathogens [48]. Assuming that the reason for adverse effects may be an increase of such opportunistic pathogens from *Escherichia-Shigella* spp., later reduction of adverse reactions could be associated with specific characteristics of these taxonomic groups. *Escherichia* and *Shigella* are two closely related genera that share bioenergetic mechanisms that allow them to fill a specific niche in the gut microbiome ecosystem [49]. Despite a competitive advantage as a facultative anaerobe, the population of *Escherichia coli* is known to be dependent on substrates provided by polysaccharide-degrading anaerobes [50]. Thus, the rapid initial growth might be terminated by the lack of mono- and disaccharides caused by reduced abundance of anaerobic mucus-associated taxonomic groups and increased competition for the limited amount of energy substrates within the taxa. Also, T2D therapy accompanying a specific diet with reduced amount of simple carbohydrates [51] may play a role in limiting the amount of substrate. That could lead to further stabilization of the microbial ecosystem and recovery of metformin tolerance. Nevertheless, the initial side effects are the main reason for metformin discontinuation in 5% of patients [37]. Our results show an increased initial presence of *Escherichia-Shigella* spp. in the samples taken before metformin administration from the participants later experiencing side effects versus those without side effects (*Escherichia-Shigella* spp. below detectable limits). Development and implementation of a test for the presence of pathogens prior to metformin administration may allow stratification of treatment strategies (e.g. dose reduction or use of slow release forms) in high-risk patients.

A limitation of the present approach is the fact that analysis of *16S rRNA* sequencing results merge together various *Escherichia-Shigella* spp. species and strains with a wide spectrum of functions, effects, and ways of interaction [52]. Therefore, further metagenomic analysis in a longitudinal study providing information on gene richness, composition, and metabolic pathways could give deeper taxonomic and functional insight into the specificity of metformin-induced changes.

In addition, the sample collection procedure that involved temporary storage at room temperature prior to freezing can be seen as a possible limitation of the study. However, it has been shown in various studies that such approach does not significantly alter the microbiome composition if the storage is up to 24 hours [23, 24].

Despite the fact that it is still hard to distinguish whether dysbiosis of the gut microbiome is the cause or consequence of T2D and a specter of various other diseases, many therapeutic effects of gut microbiome modulation have been proven already [14, 53, 54]. It has been suggested that, despite induction of GI associated side effects, metformin may also exert its positive effects through its capability to modulate the gut microbiome. The strongest observable and specific effect of metformin in our study was the reduction in abundance of the family *Peptostreptococcaceae* and three genera within it. Members of this family, in principle, have been associated with compromised health–one of the most convincing examples being *Clostridium difficile*. Increased abundance of *Peptostreptococcaceae* has also been associated with such conditions as non-alcoholic fatty liver disease [55], ulcerative colitis [56], and colorectal cancer [57], as well as with reduced lifespan [58]. In addition, reduced abundance of this family has been found in mice fed with a low-fat diet [59] or with calorie restrictions [58]. Interestingly, both families, significantly decreased by metformin, have been described to show similar response tendencies in various studies. Both *Peptostreptococcaceae* and *Clostridiaceae_1* possibly mediate the effect of eugenol treatment on mucus production in mice [60] and may be associated with dietary protein restriction induced improvement of ileal barrier function in pigs [61].

In the context of T2D or metformin therapy, the family *Peptostreptococcaceae* in general has not been described before, but previous studies have found significantly reduced abundance of one genus within it–*Intestinibacter* spp.–associated with metformin treatment [11, 12]. The functional role of this genus is still unclear, as it has been defined only recently [62]. We did not observe any statistically significant changes in the abundance of this genus that might be explained by analysis of healthy individuals in our study group.

The possibly controversial role of these taxa could be explained by potential differences in genera and species composition within these families between human and animal gut microbiomes. Overall, these changes in taxonomic units show that metformin may have beneficial effects through modification of possibly unfavorable human gut microbiome composition.

Unlike previous studies, we did not observe a significant increase in abundance of *Akkermansia* spp. after correction. One of the reasons may be the low prevalence of this genus in our study group that can be explained by population, age, or disease status based differences when comparing to other studies.

Another intriguing question is the mechanism of how metformin modifies the gut microbiome. Recently, it has been shown that metformin has a direct effect on some, but not all of the gut microbiome bacteria, that was demonstrated by decreased growth in the presence of metformin *in vitro* [12]. It is not yet clear whether these direct effects of metformin are sufficient to explain the broad range of taxa affected in gut. Alternatively, the microbiome changes at least in part can be the result of systemic effects of metformin on the host (e.g. altered enterohepatic circulation of bile acids and salts) as suggested in McCreight et al. (2016) [7]. Our data, however, show rapid metformin-induced effects, and thus are in favor of the direct action of metformin, although this has to be proven using additional *in vitro* studies.

In conclusion, we were able to present direct evidence of effects of metformin on the gut microbiome in humans using prospective study, and associate these changes with metformin side effects. As this is an exploratory study without a placebo control arm, it would require additional randomized controlled trial in order to reach definitive conclusions. Nevertheless, our results indicate the possibility of developing a personalized approach in metformin therapy by pre-screening gut microbiota for abundance of opportunistic pathogens, followed by adjusted therapeutic strategies in patients with higher risk of developing side effects.

## Supporting information

S1 Text List of inclusion/exclusion criteria. (DOCX)

S2 Text CONSORT checklist. (DOCX)

S3 Text Clinical trial information. (PDF)

S1 Table Summary of the registered side effects during the metformin use. (DOCX)

S2 Table Summary of 7-day food record during the metformin treatment. (DOCX)

S1 Fig Taxonomic composition in all samples at family level. Plot visualizes the high interindividual diversity represented by most abundant taxonomic groups at family level. Each individual is marked with a personal identification code.
(PDF)

## References

[1] American Diabetes Association. Standards of medical care in diabetes -2010. Diabetes Care. 2010;33 Suppl 1:S11–61. 20042772 10.2337/dc10-S011 PMC2797382

[2] Bolen S, Feldman L, Vassy J, Wilson L, Yeh HC, Marinopoulos S, et al Systematic review: comparative effectiveness and safety of oral medications for type 2 diabetes mellitus. Ann Intern Med. 2007;147(6):386–99. 17638715 10.7326/0003-4819-147-6-200709180-00178

[3] Inzucchi SE, Bergenstal RM, Buse JB, Diamant M, Ferrannini E, Nauck M, et al Management of hyperglycemia in type 2 diabetes, 2015: a patient-centered approach: update to a position statement of the American Diabetes Association and the European Association for the Study of Diabetes. Diabetes Care. 2015;38(1):140–9. 10.2337/dc14-2441 25538310

[4] Fung CS, Wan EY, Wong CK, Jiao F, Chan AK. Effect of metformin monotherapy on cardiovascular diseases and mortality: a retrospective cohort study on Chinese type 2 diabetes mellitus patients. Cardiovasc Diabetol. 2015;14:137 10.1186/s12933-015-0304-2 26453464 PMC4600251

[5] Nasri H, Rafieian-Kopaei M. Metformin: Current knowledge. J Res Med Sci. 2014;19(7):658–64. 25364368 PMC4214027

[6] Bonora E, Cigolini M, Bosello O, Zancanaro C, Capretti L, Zavaroni I, et al Lack of effect of intravenous metformin on plasma concentrations of glucose, insulin, C-peptide, glucagon and growth hormone in non-diabetic subjects. Curr Med Res Opin. 1984;9(1):47–51. 10.1185/03007998409109558 6373159

[7] McCreight LJ, Bailey CJ, Pearson ER. Metformin and the gastrointestinal tract. Diabetologia. 2016;59(3):426–35. 10.1007/s00125-015-3844-9 26780750 PMC4742508

[8] Graham GG, Punt J, Arora M, Day RO, Doogue MP, Duong JK, et al Clinical pharmacokinetics of metformin. Clin Pharmacokinet. 2011;50(2):81–98. 10.2165/11534750-000000000-00000 21241070

[9] Buse JB, DeFronzo RA, Rosenstock J, Kim T, Burns C, Skare S, et al The Primary Glucose-Lowering Effect of Metformin Resides in the Gut, Not the Circulation: Results From Short-term Pharmacokinetic and 12-Week Dose-Ranging Studies. Diabetes Care. 2016;39(2):198–205. 10.2337/dc15-0488 26285584

[10] de la Cuesta-Zuluaga J, Mueller NT, Corrales-Agudelo V, Velásquez-Mejía EP, Carmona JA, Abad JM, et al Metformin Is Associated With Higher Relative Abundance of Mucin-Degrading Akkermansia muciniphila and Several Short-Chain Fatty Acid–Producing Microbiota in the Gut. Diabetes Care. 2016. 10.2337/dc16-1324 27999002

[11] Forslund K, Hildebrand F, Nielsen T, Falony G, Le Chatelier E, Sunagawa S, et al Disentangling type 2 diabetes and metformin treatment signatures in the human gut microbiota. Nature. 2015;528(7581):262–6. 10.1038/nature15766 26633628 PMC4681099

[12] Wu H, Esteve E, Tremaroli V, Khan MT, Caesar R, Manneras-Holm L, et al Metformin alters the gut microbiome of individuals with treatment-naive type 2 diabetes, contributing to the therapeutic effects of the drug. Nat Med. 2017. 10.1038/nm.4345 28530702

[13] Lee H, Ko G. Effect of metformin on metabolic improvement and gut microbiota. Appl Environ Microbiol. 2014;80(19):5935–43. 10.1128/AEM.01357-14 25038099 PMC4178684

[14] Shin NR, Lee JC, Lee HY, Kim MS, Whon TW, Lee MS, et al An increase in the Akkermansia spp. population induced by metformin treatment improves glucose homeostasis in diet-induced obese mice. Gut. 2014;63(5):727–35. 10.1136/gutjnl-2012-303839 23804561

[15] Napolitano A, Miller S, Nicholls AW, Baker D, Van Horn S, Thomas E, et al Novel gut-based pharmacology of metformin in patients with type 2 diabetes mellitus. PLoS One. 2014;9(7):e100778 10.1371/journal.pone.0100778 24988476 PMC4079657

[16] Burton JH, Johnson M, Johnson J, Hsia DS, Greenway FL, Heiman ML. Addition of a Gastrointestinal Microbiome Modulator to Metformin Improves Metformin Tolerance and Fasting Glucose Levels. J Diabetes Sci Technol. 2015. 10.1177/1932296815577425 PMC4525649 25802471

[17] Zhang X, Zhao Y, Xu J, Xue Z, Zhang M, Pang X, et al Modulation of gut microbiota by berberine and metformin during the treatment of high-fat diet-induced obesity in rats. Sci Rep. 2015;5:14405 10.1038/srep14405 26396057 PMC4585776

[18] Maniar K, Moideen A, Bhattacharyya R, Banerjee D. Metformin exerts anti-obesity effect via gut microbiome modulation in prediabetics: A hypothesis. Med Hypotheses. 2017;104:117–20. 10.1016/j.mehy.2017.06.001 28673568

[19] Barzilai N, Crandall JP, Kritchevsky SB, Espeland MA. Metformin as a Tool to Target Aging. Cell Metab. 2016;23(6):1060–5. 10.1016/j.cmet.2016.05.011 27304507 PMC5943638

[20] Evans JM, Donnelly LA, Emslie-Smith AM, Alessi DR, Morris AD. Metformin and reduced risk of cancer in diabetic patients. BMJ. 2005;330(7503):1304–5. 10.1136/bmj.38415.708634.F7 15849206 PMC558205

[21] Moghetti P, Castello R, Negri C, Tosi F, Perrone F, Caputo M, et al Metformin effects on clinical features, endocrine and metabolic profiles, and insulin sensitivity in polycystic ovary syndrome: a randomized, double-blind, placebo-controlled 6-month trial, followed by open, long-term clinical evaluation. J Clin Endocrinol Metab. 2000;85(1):139–46. 10.1210/jcem.85.1.6293 10634377

[22] Rovite V, Wolff-Sagi Y, Zaharenko L, Nikitina-Zake L, Grens E, Klovins J. Genome Database of the Latvian Population (LGDB): Design, Goals, and Primary Results. J Epidemiol. 2018. 10.2188/jea.JE20170079 PMC6048300 29576601

[23] Cardona S, Eck A, Cassellas M, Gallart M, Alastrue C, Dore J, et al Storage conditions of intestinal microbiota matter in metagenomic analysis. BMC Microbiol. 2012;12:158 10.1186/1471-2180-12-158 22846661 PMC3489833

[24] Tedjo DI, Jonkers DM, Savelkoul PH, Masclee AA, van Best N, Pierik MJ, et al The effect of sampling and storage on the fecal microbiota composition in healthy and diseased subjects. PLoS One. 2015;10(5):e0126685 10.1371/journal.pone.0126685 26024217 PMC4449036

[25] Milani C, Hevia A, Foroni E, Duranti S, Turroni F, Lugli GA, et al Assessing the fecal microbiota: an optimized ion torrent 16S rRNA gene-based analysis protocol. PLoS One. 2013;8(7):e68739 10.1371/journal.pone.0068739 23869230 PMC3711900

[26] Schloss PD, Westcott SL, Ryabin T, Hall JR, Hartmann M, Hollister EB, et al Introducing mothur: open-source, platform-independent, community-supported software for describing and comparing microbial communities. Appl Environ Microbiol. 2009;75(23):7537–41. 10.1128/AEM.01541-09 19801464 PMC2786419

[27] Quast C, Pruesse E, Yilmaz P, Gerken J, Schweer T, Yarza P, et al The SILVA ribosomal RNA gene database project: improved data processing and web-based tools. Nucleic Acids Res. 2013;41(Database issue):D590–6. 10.1093/nar/gks1219 23193283 PMC3531112

[28] Edgar RC, Haas BJ, Clemente JC, Quince C, Knight R. UCHIME improves sensitivity and speed of chimera detection. Bioinformatics. 2011;27(16):2194–200. 10.1093/bioinformatics/btr381 21700674 PMC3150044

[29] Wang Q, Garrity GM, Tiedje JM, Cole JR. Naive Bayesian classifier for rapid assignment of rRNA sequences into the new bacterial taxonomy. Appl Environ Microbiol. 2007;73(16):5261–7. 10.1128/AEM.00062-07 17586664 PMC1950982

[30] Segata N, Izard J, Waldron L, Gevers D, Miropolsky L, Garrett WS, et al Metagenomic biomarker discovery and explanation. Genome Biol. 2011;12(6):R60 10.1186/gb-2011-12-6-r60 21702898 PMC3218848

[31] Shannon CE. A Mathematical Theory of Communication. Bell System Technical Journal. 1948;27(3):379–423.

[32] Goodrich JK, Di Rienzi SC, Poole AC, Koren O, Walters WA, Caporaso JG, et al Conducting a microbiome study. Cell. 2014;158(2):250–62. 10.1016/j.cell.2014.06.037 25036628 PMC5074386

[33] Duvallet C, Gibbons SM, Gurry T, Irizarry RA, Alm EJ. Meta-analysis of gut microbiome studies identifies disease-specific and shared responses. Nat Commun. 2017;8(1):1784 10.1038/s41467-017-01973-8 29209090 PMC5716994

[34] Gupta A, Bisht B, Dey CS. Peripheral insulin-sensitizer drug metformin ameliorates neuronal insulin resistance and Alzheimer's-like changes. Neuropharmacology. 2011;60(6):910–20. 10.1016/j.neuropharm.2011.01.033 21277873

[35] Lily M, Godwin M. Treating prediabetes with metformin: systematic review and meta-analysis. Can Fam Physician. 2009;55(4):363–9. 19366942 PMC2669003

[36] Hoffmann IS, Roa M, Torrico F, Cubeddu LX. Ondansetron and metformin-induced gastrointestinal side effects. Am J Ther. 2003;10(6):447–51. 14624284 10.1097/00045391-200311000-00012

[37] Kirpichnikov D, McFarlane SI, Sowers JR. Metformin: an update. Ann Intern Med. 2002;137(1):25–33. 12093242 10.7326/0003-4819-137-1-200207020-00009

[38] Dujic T, Causevic A, Bego T, Malenica M, Velija-Asimi Z, Pearson ER, et al Organic cation transporter 1 variants and gastrointestinal side effects of metformin in patients with Type 2 diabetes. Diabet Med. 2016;33(4):511–4. 10.1111/dme.13040 26605869 PMC5064645

[39] Florez H, Luo J, Castillo-Florez S, Mitsi G, Hanna J, Tamariz L, et al Impact of metformin-induced gastrointestinal symptoms on quality of life and adherence in patients with type 2 diabetes. Postgrad Med. 2010;122(2):112–20. 10.3810/pgm.2010.03.2128 20203462

[40] Amato SM, Orman MA, Brynildsen MP. Metabolic control of persister formation in Escherichia coli. Mol Cell. 2013;50(4):475–87. 10.1016/j.molcel.2013.04.002 23665232

[41] Touchon M, Hoede C, Tenaillon O, Barbe V, Baeriswyl S, Bidet P, et al Organised genome dynamics in the Escherichia coli species results in highly diverse adaptive paths. PLoS Genet. 2009;5(1):e1000344 10.1371/journal.pgen.1000344 19165319 PMC2617782

[42] Ticinesi A, Milani C, Lauretani F, Nouvenne A, Mancabelli L, Lugli GA, et al Gut microbiota composition is associated with polypharmacy in elderly hospitalized patients. Sci Rep. 2017;7(1):11102 10.1038/s41598-017-10734-y 28894183 PMC5593887

[43] Francino MP. Antibiotics and the Human Gut Microbiome: Dysbioses and Accumulation of Resistances. Front Microbiol. 2015;6:1543 10.3389/fmicb.2015.01543 26793178 PMC4709861

[44] Milani C, Ticinesi A, Gerritsen J, Nouvenne A, Lugli GA, Mancabelli L, et al Gut microbiota composition and Clostridium difficile infection in hospitalized elderly individuals: a metagenomic study. Sci Rep. 2016;6:25945 10.1038/srep25945 27166072 PMC4863157

[45] Morton ER, Lynch J, Froment A, Lafosse S, Heyer E, Przeworski M, et al Variation in Rural African Gut Microbiota Is Strongly Correlated with Colonization by Entamoeba and Subsistence. PLoS Genet. 2015;11(11):e1005658 10.1371/journal.pgen.1005658 26619199 PMC4664238

[46] Haupt E, Knick B, Koschinsky T, Liebermeister H, Schneider J, Hirche H. Oral antidiabetic combination therapy with sulphonylureas and metformin. Diabete Metab. 1991;17(1 Pt 2):224–31. 1936481

[47] Howlett HC, Bailey CJ. A risk-benefit assessment of metformin in type 2 diabetes mellitus. Drug Saf. 1999;20(6):489–503. 10392666 10.2165/00002018-199920060-00003

[48] Sims GE, Kim SH. Whole-genome phylogeny of Escherichia coli/Shigella group by feature frequency profiles (FFPs). Proc Natl Acad Sci U S A. 2011;108(20):8329–34. 10.1073/pnas.1105168108 21536867 PMC3100984

[49] Johnson JR. Shigella and Escherichia coli at the crossroads: machiavellian masqueraders or taxonomic treachery? J Med Microbiol. 2000;49(7):583–5. 10.1099/0022-1317-49-7-583 10882082

[50] Jones SA, Chowdhury FZ, Fabich AJ, Anderson A, Schreiner DM, House AL, et al Respiration of Escherichia coli in the mouse intestine. Infect Immun. 2007;75(10):4891–9. 10.1128/IAI.00484-07 17698572 PMC2044527

[51] Asif M. The prevention and control the type-2 diabetes by changing lifestyle and dietary pattern. J Educ Health Promot. 2014;3:1 10.4103/2277-9531.127541 24741641 PMC3977406

[52] Gao YD, Zhao Y, Huang J. Metabolic modeling of common Escherichia coli strains in human gut microbiome. Biomed Res Int. 2014;2014:694967 10.1155/2014/694967 25126572 PMC4122010

[53] Mattila E, Uusitalo-Seppala R, Wuorela M, Lehtola L, Nurmi H, Ristikankare M, et al Fecal transplantation, through colonoscopy, is effective therapy for recurrent Clostridium difficile infection. Gastroenterology. 2012;142(3):490–6. 10.1053/j.gastro.2011.11.037 22155369

[54] Rossi O, van Berkel LA, Chain F, Tanweer Khan M, Taverne N, Sokol H, et al Faecalibacterium prausnitzii A2-165 has a high capacity to induce IL-10 in human and murine dendritic cells and modulates T cell responses. Sci Rep. 2016;6:18507 10.1038/srep18507 26725514 PMC4698756

[55] Jiang W, Wu N, Wang X, Chi Y, Zhang Y, Qiu X, et al Dysbiosis gut microbiota associated with inflammation and impaired mucosal immune function in intestine of humans with non-alcoholic fatty liver disease. Sci Rep. 2015;5:8096 10.1038/srep08096 25644696 PMC4314632

[56] Lavelle A, Lennon G, O'Sullivan O, Docherty N, Balfe A, Maguire A, et al Spatial variation of the colonic microbiota in patients with ulcerative colitis and control volunteers. Gut. 2015;64(10):1553–61. 10.1136/gutjnl-2014-307873 25596182 PMC4602252

[57] Chen W, Liu F, Ling Z, Tong X, Xiang C. Human intestinal lumen and mucosa-associated microbiota in patients with colorectal cancer. PLoS One. 2012;7(6):e39743 10.1371/journal.pone.0039743 22761885 PMC3386193

[58] Zhang C, Li S, Yang L, Huang P, Li W, Wang S, et al Structural modulation of gut microbiota in life-long calorie-restricted mice. Nat Commun. 2013;4:2163 10.1038/ncomms3163 23860099 PMC3717500

[59] Cox LM, Cho I, Young SA, Anderson WH, Waters BJ, Hung SC, et al The nonfermentable dietary fiber hydroxypropyl methylcellulose modulates intestinal microbiota. FASEB J. 2013;27(2):692–702. 10.1096/fj.12-219477 23154883 PMC3545536

[60] Wlodarska M, Willing BP, Bravo DM, Finlay BB. Phytonutrient diet supplementation promotes beneficial Clostridia species and intestinal mucus secretion resulting in protection against enteric infection. Sci Rep. 2015;5:9253 10.1038/srep09253 25787310 PMC4365398

[61] Fan P, Liu P, Song P, Chen X, Ma X. Moderate dietary protein restriction alters the composition of gut microbiota and improves ileal barrier function in adult pig model. Sci Rep. 2017;7:43412 10.1038/srep43412 28252026 PMC5333114

[62] Gerritsen J, Fuentes S, Grievink W, van Niftrik L, Tindall BJ, Timmerman HM, et al Characterization of Romboutsia ilealis gen. nov., sp. nov., isolated from the gastro-intestinal tract of a rat, and proposal for the reclassification of five closely related members of the genus Clostridium into the genera Romboutsia gen. nov., Intestinibacter gen. nov., Terrisporobacter gen. nov. and Asaccharospora gen. nov. Int J Syst Evol Microbiol. 2014;64(Pt 5):1600–16. 10.1099/ijs.0.059543-0 24480908

