# Supplementary material for: Association of metformin administration with gut microbiome dysbiosis in healthy volunteers
Source: PLoS One. 2018 Sep 27;13(9):e0204317. doi: 10.1371/journal.pone.0204317 (PMC6160085; doi:10.1371/journal.pone.0204317)
Supplement: S1 Text — (DOCX) [file pone.0204317.s001.docx]

Supplementary Text S1 - List of inclusion/exclusion criteria

- 1. Inclusion criteria:
  2. Healthy individual (1) who at the moment of inclusion does not have known diseases that could affect the result of the study, (2) whose body characteristics are within the healthy reference interval (e.g., BMI range is 18.5 – 29.9), (3) whose mental state allows to understand the study, and to give legal agreement to participation, (4) whose physical state allows to comply with the needs of study protocol.
  3. Age: 18 – 64 years.
  4. European origin.
  5. Both males and females with reproductive potential match the contraception requirements of the study protocol.
  6. Before starting any of the procedures related to the study, the written consent of the participant has been received.
  7. Exclusion criteria:
  8. Hypersensitivity to any of the excipients in *Metforal* 850mg.
  9. Use of any medicaments that are not compatible with *Metforal* 850mg therapy (according to *Metforal* description).
  10. Female who is pregnant or lactating.
  11. Diagnosed type 1 or type 2 diabetes mellitus, pancreatogenic diabetes, impaired glucose tolerance (evaluated by HbA1c and fasting glucose levels).
  12. Polycystic ovary syndrome.
  13. Chronical gastrointestinal, oncological, or autoimmune diseases.
  14. Renal failure or dysfunction (evaluated by glomerular filtration rate - Cockcroft-Gault formula).
  15. Liver dysfunction (alanine aminotransferase (ALAT) results are not in the reference interval) or alcoholism.
  16. Acute conditions with possible effect on kidney functions: dehydration, severe infection, shock.
  17. Acute or chronical diseases that could cause tissue hypoxia, e.g., heart or breathing failure, recent myocardial infarct, shock.
  18. Diarrhea in the time period of one weeks before the study.
  19. Previous long term use of metformin.
  20. Use of any of the following medications in the past two months: antibiotics, pharmaceutical-grade probiotics, proton pump inhibitors (e.g. omeprazole, lansoprazole, pantoprazole, etc.), immunosuppressive drugs (methotrexate, etc.), corticosteroids (e.g. cortisone, hydrocortisone, prednisolone, etc.).
  21. Concurrently to the study, any radiologic procedure involving intravascular administration of iodinated contrast materials have been planned.
