# Supplementary material for: Association of metformin administration with gut microbiome dysbiosis in healthy volunteers
Source: PLoS One. 2018 Sep 27;13(9):e0204317. doi: 10.1371/journal.pone.0204317 (PMC6160085; doi:10.1371/journal.pone.0204317)
Supplement: S1 Table — (DOCX) [file pone.0204317.s004.docx]

S1 Table. Summary of the registered side effects during the metformin use.

S1.1 Table. Side effect registration during the study

| Side effects | Day 1 | | Day 2 | | Day 3 | | Day 4 | | Day 5 | | | Day 6 | | Day 7 | | Total number of individuals | |
| --- | --- | --- | --- | --- | --- | --- | --- | --- | --- | --- | --- | --- | --- | --- | --- | --- | --- |
|  | N | % | N | % | N | % | N | % | N | % | | N | % | N | % | N | % |
| Nausea | 3 | 16.7 | 4 | 22.2 | 5 | 27.8 | 6 | 33.3 | 3 | 16.7 | | 3 | 16.7 | 3 | 16.7 | 10 | 55.6 |
| Weakness | 1 | 5.6 | 1 | 5.6 | 2 | 11.1 | 3 | 16.7 | 1 | 5.6 | | 0 | 0.0 | 0 | 0.0 | 3 | 16.7 |
| Belching | 2 | 11.1 | 2 | 11.1 | 2 | 11.1 | 2 | 11.1 | 3 | 16.7 | | 1 | 5.6 | 1 | 5.6 | 4 | 22.2 |
| Meteorism | 2 | 11.1 | 5 | 27.8 | 7 | 38.9 | 7 | 38.9 | 4 | 22.2 | | 1 | 5.6 | 2 | 11.1 | 8 | 44.4 |
| Loose stools 1-2 times/day | 4 | 22.2 | 2 | 11.1 | 5 | 27.8 | 2 | 11.1 | 1 | 5.6 | | 1 | 5.6 | 0 | 0.0 | 7 | 38.9 |
| Diarrhea | 0 | 0.0 | 2 | 11.1 | 2 | 11.1 | 1 | 5.6 | 2 | 11.1 | | 0 | 0.0 | 1 | 5.6 | 2 | 11.1 |
| Vomiting | 0 | 0.0 | 0 | 0.0 | 0 | 0.0 | 0 | 0.0 | 0 | 0.0 | | 0 | 0.0 | 0 | 0.0 | 0 | 0.0 |
| Decreased appetite | 1 | 5.6 | 1 | 5.6 | 2 | 11.1 | 2 | 11.1 | 2 | 11.1 | | 2 | 11.1 | 2 | 11.1 | 3 | 16.7 |
| Total number of individuals with side effects (m – mild, s – severe) | m – 6  s ‑ 4 | 33.3  22.2 | m – 10  s ‑ 4 | 55.6  22.2 | m – 8  s ‑ 7 | 44.4  38.9 | m – 8  s ‑ 3 | 44.4  16.7 | m – 10  s ‑ 3 | | 55.6  16.7 | m – 5  s ‑ 1 | 37.8  5.6 | m – 6  s ‑ 1 | 33.3  5.6 | m – 6  s ‑ 9 | 33.3  50.0 |

**S1.2. Table. Observed side effects for each individual.**

| Individual | Day | | | | | | | | | | | |
| --- | --- | --- | --- | --- | --- | --- | --- | --- | --- | --- | --- | --- |
|  | 1 | | 2 | | 3 | | 4 | | 5 | | 6 | 7 |
| Subject_1 |  | |  | |  | |  | |  | |  |  |
| Subject_2 |  | |  | |  | |  | |  | |  |  |
| Subject_3 |  | |  | |  | |  | |  | |  |  |
| Subject_4 |  | |  |  |  |  |  | |  | |  |  |
| Subject_5 |  |  |  | |  |  |  | |  |  |  |  |
| Subject_6 |  | |  | |  | |  |  |  | |  |  |
| Subject_7 |  | |  | |  | |  | |  | |  |  |
| Subject_8 |  | |  |  |  |  |  |  |  |  |  |  |
| Subject_9 |  | |  | |  | |  | |  | |  |  |
| Subject_10 |  | |  | |  | |  | |  | |  |  |
| Subject_11 |  | |  | |  |  |  | |  | | - | - |
| Subject_12 |  | |  | |  | |  | |  | |  |  |
| Subject_13 |  | |  | |  | |  | |  | |  |  |
| Subject_14 |  | |  | |  | |  | |  | |  |  |
| Subject_15 |  |  |  |  |  |  |  |  |  |  |  |  |
| Subject_16 |  | |  | |  | |  | |  | |  |  |
| Subject_17 |  | |  | |  | |  | |  | |  |  |
| Subject_18 |  | |  | |  | |  | |  | |  |  |

Different colors represent the three defined side effect groups: (1) Green – no side effects; (2) Yellow – mild side effects; (3) Red – severe side effects. “-“ represents the case of early withdrawal from the study and the days of not participating in the trial. Days containing both yellow and red colors describe the presence of both mild and severe side effects.
