# Supplementary material for: Association of metformin administration with gut microbiome dysbiosis in healthy volunteers
Source: PLoS One. 2018 Sep 27;13(9):e0204317. doi: 10.1371/journal.pone.0204317 (PMC6160085; doi:10.1371/journal.pone.0204317)
Supplement: S2 Table — (DOCX) [file pone.0204317.s005.docx]

**S2 Table. Summary of 7-day food record during the metformin treatment.**

| **ID** | **Food group, %** | | | | | | | | | | |
| --- | --- | --- | --- | --- | --- | --- | --- | --- | --- | --- | --- |
|  | **Milk and dairy products** | **Vegetables** | **Fruits** | **Meat and its products** | **Fish** | **Croppers** | **Nuts and seeds** | **Fat** | **Snacks** | **Sweetened drinks** | **Alcohol** |
| MF0006 | 11 | 2 | 1 | 7 | 0 | 41 | 0 | 19 | 4 | 0 | 15 |
| MF0007 | 29 | 5 | 0 | 3 | 0 | 18 | 1 | 14 | 2 | 2 | 26 |
| MF0008 | 6 | 8 | 8 | 5 | 0 | 19 | 3 | 29 | 0 | 0 | 22 |
| MF0012 | 7 | 5 | 4 | 7 | 0 | 5 | 0 | 32 | 3 | 15 | 21 |
| MF0015 | 8 | 4 | 17 | 1 | 0 | 19 | 0 | 35 | 0 | 0 | 15 |
| MF0016 | 7 | 7 | 12 | 5 | 2 | 22 | 0 | 18 | 1 | 0 | 27 |
| MF0017 | 3 | 7 | 10 | 7 | 3 | 17 | 0 | 25 | 1 | 0 | 26 |
| MF0018 | 5 | 10 | 8 | 8 | 0 | 20 | 0 | 27 | 1 | 0 | 21 |
| MF0020 | 10 | 6 | 6 | 4 | 2 | 30 | 1 | 16 | 7 | 0 | 17 |
| MM0004 | 14 | 4 | 0 | 10 | 2 | 19 | 0 | 26 | 5 | 1 | 19 |
| MM0005 | 17 | 5 | 10 | 10 | 1 | 18 | 0 | 23 | 0 | 0 | 16 |
| MM0007 | 7 | 8 | 7 | 10 | 1 | 10 | 0 | 40 | 1 | 1 | 14 |
| MM0008 | 9 | 5 | 2 | 8 | 3 | 22 | 0 | 15 | 5 | 0 | 32 |
| MM0009 | 7 | 5 | 3 | 6 | 1 | 17 | 0 | 30 | 7 | 0 | 23 |
| MM0010 | 7 | 6 | 9 | 8 | 3 | 8 | 0 | 29 | 2 | 4 | 24 |
| MM0011 | 12 | 10 | 5 | 10 | 1 | 30 | 0 | 7 | 0 | 4 | 19 |
| MM0013 | 4 | 8 | 5 | 11 | 1 | 18 | 1 | 11 | 0 | 4 | 37 |
| MM0014 | 11 | 9 | 7 | 6 | 1 | 12 | 0 | 35 | 0 | 0 | 20 |
