# Supplementary figures and images for: Association of metformin administration with gut microbiome dysbiosis in healthy volunteers
Source: PLoS One. 2018 Sep 27;13(9):e0204317. doi: 10.1371/journal.pone.0204317 (PMC6160085; doi:10.1371/journal.pone.0204317)

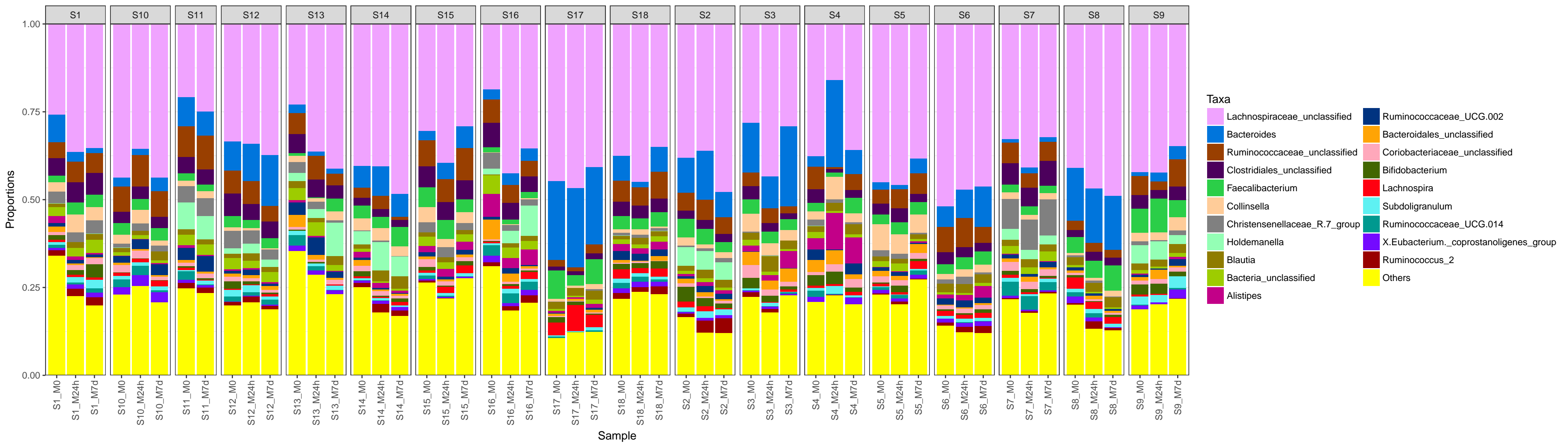

Supplement: S1 Fig — Plot visualizes the high interindividual diversity represented by most abundant taxonomic groups at family level. Each individual is marked with a personal identification code. (PDF) [file pone.0204317.s006.pdf]
